# Qualitative analysis of the main pillars of the Swimming Nation Program

**DOI:** 10.3389/fspor.2026.1739939

**Published:** 2026-04-10

**Authors:** Gergely Martony, Péter Szájer, Nikoletta Nagy, Csaba Ökrös, István Csáki

**Affiliations:** 1 Department of Sports Games, Hungarian University of Sports Science, Budapest, Hungary; 2 School of Doctoral Studies, Hungarian University of Sports Science, Budapest, Hungary; 3 Department of Swimming and Water Sports, Hungarian University of Sports Science, Budapest, Hungary

**Keywords:** program evaluation, qualitative research, sport development, sport policy, youth development

## Abstract

This study explores the structure and operation of the Swimming Nation Program using a qualitative approach based on document analysis and expert interviews (*N* = 9). The analysis focuses on four central components of the program: infrastructure, curriculum, instructors, and logistics. The findings indicate that the program functions as a coordinated national system that enables broad access to swimming education through its integration into the public education framework. At the same time, several structural limitations were identified, particularly in relation to the uneven distribution of facilities and the availability of qualified instructors. A total of 122 facilities met the criteria for participation, revealing clear regional differences in access. Logistics emerged as a key element that helps mitigate these disparities by ensuring that children can reach swimming facilities even in areas with limited infrastructure. In addition to its educational role, the program also contributes to expanding the talent pool by introducing a large number of children to swimming at an early age. Overall, the study highlights the importance of coordinated infrastructure, human resources, and organizational processes in the effective implementation of large-scale sport development programs.

## Introduction

### The relationship between youth development programs and sporting success

In Hungary, youth development is a crucial factor when it comes to achieving success in sports (1). Development programs support the growth of sporting talent in Hungary. One of the first to be mentioned is the Herakles Champion Program, which started in 2001. Initially launched with 13 sports, it has since expanded to 37 sports. This project exclusively provided opportunities and support for selected athletes between the ages of 14–18 to develop in their respective sports.

Years later, the Herakles Star Program was initiated, which has been supporting athletes up to the age of 23 since 2006. Its primary goals were to keep athletes on track, manage their careers, ensure international competition, and integrate them into the adult framework. The program also provided remuneration for various professionals, such as sports doctors and psychologists (2).

Furthermore, since 2005, the Sport XXI Youth Development Program, operating under the Herakles Champion Program, was created, targeting elementary school-aged children. Its aim was to attract as many children as possible into the system and provide equal opportunities for everyone (2). The professional team behind it wanted to create a broader vision program that goes beyond youth development, focusing primarily on the physical development of children under 14, daily exercise, and fostering a lifelong love for sports (3).

The concept of the sports school has existed since 1997, following the establishment of the National Association of Sports Schools (4). The advantage of sports schools, as public education institutions, is that youth development is organized in a structured way. There are two types: non-public education (club-type) and public education-type schools (2). A previous study indicated that the biggest difference between sports schools and general elementary schools is the time spent on sports, which increases even more at the high school level (5).

The Herakles program established the notion that organized management is necessary for talented athletes to come to the forefront in sports (6). It is now widely accepted that the nation's greatest talents, capable of achieving significant results in their sports, often emerge from these programs. The success of the program could already be quantified at the 2008 Beijing Olympics, where 37% of the 170 participating athletes, or about 63 individuals, were part of the “Herakles team.” This number increased significantly by the time of the 2012 London Olympics, with 103 out of 148 competitors (70%) being members of the program (7).

When discussing youth development programs, the Long-Term Athlete Development (LTAD) model, associated with István Balyi, must be mentioned. This program emphasizes planning within preparation, starting from early childhood up to the national team level and covering the youth age group (8). This long-term development model highlights the importance of focusing on athletes' sensitive developmental periods, age-specific characteristics, and quality competition systems for proper improvement (9).

### The impact of swimming on health education and a healthy lifestyle

Swimming is a sport that, besides its effects on the body, plays a significant role in personality development (10). Its positive health-enhancing impact extends to the muscular, nervous, and skeletal systems, as well as various organs and organ systems. It stimulates circulation, refreshes the body, and positively affects the autonomic nervous system (11). Additionally, it boosts metabolism and influences cardiovascular function. What sets swimming apart from other sports is that it is performed in a different medium—water—making it a recommended recreational activity (12, 13). Swimming is the only sport in the world that has a beneficial effect on the respiratory system since breathing must be coordinated with the different swimming styles and practises (10). As such, it has a preventive effect against asthmatic symptoms, which, unfortunately, are becoming more prevalent alongside allergies (11). Another major societal issue that swimming positively impacts is postural deformities. Swimming helps to strengthen the muscles supporting the spine when it comes to correcting postural problems (11). It can be stated that swimming is considered a “life-time” sport, meaning it can be practiced from early childhood through old age, either individually or with the entire family (14–19).

### Swimming education in Hungary

Following the introduction of daily physical education in 2012, there were many more opportunities to develop swimming education, which typically consisted of ten 45-minute sessions per group annually (20). In 2013, with government support, the Hungarian Swimming Federation (hereafter: HSF) launched the “Every Child Should Learn to Swim” program, which provided swimming lessons through sports clubs. In physical education, the teaching of self-rescue and lifesaving skills has also gained prominence, primarily as part of efforts to enhance safety and reduce the risk of drowning (21). Consequently, the importance of swimming extends far beyond leisure activities, as it provides essential water safety skills that are indispensable in drowning prevention (22). The program's goal was for children in schoolyear 1 and 2 to be able to swim 25 meters in one or two swimming styles by the end of the school year. To achieve this, HSF developed various teaching methodologies to assist coaches, teachers, and instructors. The guideline was to organize two 60-minute sessions per week. However, no unified teaching system was established in the program; instead, suggestions were made on how to teach each swimming styles. These suggestions were compiled into a booklet, outlining exercises and task groups for guidance. Clubs participating in the program could apply for various funding from the HSF to implement the program, which could be used for pool rental, paying specialists, or purchasing equipment. The primary goal of the program is to teach swimming, but a secondary objective is to increase the youth base (20). Finally, after evaluating previous swimming programs, experts developed and launched the most comprehensive swimming program in Hungary to date in 2019: the Swimming Nation Program (hereinafter: SNP) (23).

### Programs similar to the Swimming Nation Program

The programs presented so far primarily aim at selecting and developing sporting talents, playing a significant role in achieving domestic and international success. Next, we will highlight sports systems with national coverage that, besides building a mass base, supported the attachment with the sport. Over the past decades, numerous talent development programs have been created, primarily in team sports. Reviewing the domestic literature on youth development programs, the following can be summarized:

The OTP-MOL Bozsik Program was the first to be established in football. The program's primary goals include creating a mass base for the sport, selecting talents, and involving highly qualified coaches (24). Talent identification and development are complex processes that unfold at different levels. The OTP-MOL Bozsik Program, which was at the forefront of youth football development in Hungary, started in 2011. The program is implemented on four integrated levels (24):

- 1 Level: Bozsik children's football, aimed at building foundations and providing enjoyment through football (ages 5–12);
- 2 Level: Bozsik Evolution Program: talent selection (ages 10–14);
- 3 Level: Bozsik Elite Program: talent development (ages 15–19);
- 4 Level: Top player development.

In September 2013, the Hungarian Handball Federation (hereafter: HHF) initiated the Handball in Schools program, initially involving 50 schools, 54 physical education teachers, and 1,430 students. The program focuses primarily on elementary school students (classes 2–4), introducing them to the basic movements, techniques, and rules of handball during two weekly sessions as part of daily physical education. The program's primary goals include increasing the handball talent pool, fostering talent development, and meeting the increased physical activity needs of schoolchildren through daily physical education (25).

In tennis, the 2008 Play and Stay program, designed for children under 12, fundamentally changed the previous structure of the sport. The sessions are primarily game-based and playful, aiming to foster a love for the sport, focusing mainly on internal motivation. By emphasizing competitive attributes, children are encouraged to enjoy tennis and competition (26).

The Hungarian Athletics Federation (hereafter: HAF) was able to introduce the Kid Athletics Program as an optional curriculum for elementary students within daily physical education. Its significance is considerable because the movements in athletics, such as running, throwing, and jumping, create an effective movement culture for all children. The HAF provides a complete package for participants, making the teaching and learning of athletics more entertaining and enjoyable with colourful equipment. The main goal was to make children enjoy the movement forms of athletics (27).

The aim of this study is to examine the structure and operation of the Swimming Nation Program through the analysis of official documents and expert interviews. Particular attention is given to identifying the key components that determine the effectiveness of the program, as well as the challenges associated with its large-scale implementation. By focusing on system-level characteristics, the study seeks to provide a more comprehensive understanding of how national sport development programs function in practice.

## Material and methods

### Sample and sampling strategy

In this study, a qualitative research design was applied, combining document analysis and structured in-depth interviews. A purposive sampling strategy was used to select participants who had direct involvement in the development, implementation, or management of the Swimming Nation Program.

A total of nine experts were included in the study (*N* = 9), representing key stakeholders such as leaders of the Hungarian Swimming Federation, project managers, coordinators, and regional leaders. The aim of this sampling approach was to ensure that participants possessed comprehensive and relevant knowledge about the program's structure, operation, and challenges.

The characteristics of the participants, including their roles and the timing of the interviews, are presented in Table 1.

**Table 1:** presentation of the interview participants, source: own-edition.

Code	Classification	Type	Date
SNP1	HSF professional leader, educational methodology director	Personal	January 16, 2024.
SNP2	HSF Head coach	Personal	January 17, 2024.
SNP3	SNP Project manager	Personal	January 25, 2024.
SNP4	HSF head of finance department	Personal	January 25, 2024.
SNP5	SNP Central Coordinator	Personal	January 16, 2024.
SNP6	Regional Leader of Central Hungary (Region I)	Personal	October 26, 2023.
SNP7	Regional Leader of Central Hungary (Region II)	Personal	January 15, 2024.
SNP8	Regional Leader of Central Hungary (Region III)	Personal	February 07, 2024.
SNP9	Regional Leader of Southern Transdanubia	Online	December 13, 2023.

### Data collection

Data collection consisted of two main components: document analysis and structured in-depth interviews. In the first phase, document analysis was conducted based on materials provided by the Hungarian Swimming Federation. These documents included strategic, operational, and developmental materials related to the Swimming Nation Program, enabling a comprehensive understanding of its structure, objectives, and key pillars. In the second phase, structured in-depth interviews were conducted using a pre-defined interview guide consisting of 12 questions. The use of structured interviews ensured consistency across participants while allowing for the exploration of key thematic areas relevant to the research objectives (28). The questions focused on the conceptual foundations, operational mechanisms, infrastructural conditions, educational methodology, logistics, and perceived strengths and weaknesses of the program. All interviews were conducted either in person or online, audio-recorded, and transcribed verbatim for analysis.

### Data analysis

The collected data were analyzed using qualitative thematic analysis. First, all interview transcripts were carefully read and familiarized. In the initial phase, open coding was applied to identify recurring concepts and meaningful units within the data. In the second phase, codes were grouped into broader categories and themes. These themes were aligned with the analytical framework of the study, particularly the four core pillars of the Swimming Nation Program (infrastructure, curriculum, instructors, and logistics), as well as themes related to talent development and program evaluation.

The document analysis followed a structured approach, focusing on key dimensions such as program objectives, institutional framework, and operational mechanisms. Relevant content from the documents was systematically coded and categorized using the same thematic structure applied in the interview analysis. The analysis followed an iterative and comparative process, where findings from document analysis and interviews were continuously integrated. This approach enabled the identification of patterns, consistencies, and discrepancies across data sources.

### Validity and reliability

To ensure the credibility and trustworthiness of the research, several methodological strategies were applied. Data triangulation was implemented by combining multiple data sources, including official documents and expert interviews, allowing for cross-verification of findings. In addition, the coding and interpretation process was conducted by multiple researchers, and discrepancies were discussed until consensus was reached, enhancing the reliability of the analysis. Furthermore, detailed descriptions of the research context, participants, and analytical procedures were provided to ensure transparency and support the transferability of the findings.

## Results

Based on the analysis of documents and in-depth interviews, the findings are organized into three main thematic areas:

(1) The structural pillars of the program (infrastructure, curriculum, instructors, logistics),
(2) Talent development, and
(3) Evaluation and future directions.

### The structural pillars of the program

#### Infrastructure

The analysis revealed that infrastructure represents a fundamental enabling condition of the Swimming Nation Program, while also acting as a structural constraint. A nationwide mapping process identified facilities suitable for swimming education based on predefined criteria such as safety, pool characteristics, and year-round usability. Out of 347 assessed facilities, 122 were considered suitable for educational purposes (Table 2), highlighting a substantial gap between existing infrastructure and program requirements. This finding points to notable regional disparities, particularly affecting smaller or rural settlements where access to appropriate facilities remains limited. A key requirement is the availability of both beginner and advanced pools, which directly determines the feasibility of program implementation at the local level. Consequently, infrastructure not only supports but also shapes the territorial reach of the program.

**Table 2:** Facilities suitable for teaching swimming in Hungary. Source (32):, own-edition.

Location of swimming pools by region	Swimming pools suitable for beginner instruction			Swimming pools suitable for advanced instruction		
	Number of swimming pools (pcs)	Number of pools (pcs)	Capacity (number of people/H)	Number of swimming pools (pcs)	Number of pools (pcs)	Capacity (number of people/H)
Bács-Kiskun	4	4	111	8	12	438
Baranya	3	3	30	5	6	356
Békés	2	2	29	6	10	491
Borsod-Abaúj-Zemplén	15	15	376	18	19	1228
Budapest	26	26	717	35	43	2687
Csongrád	4	5	121	8	14	846
Fejér	4	4	96	7	9	503
Győr-Moson-Sopron	5	5	139	7	9	544
Hajdú-Bihar	7	7	144	12	15	861
Heves	4	4	114	9	12	612
Jász-Nagykun-Szolnok	3	4	77	10	15	558
Komárom-Esztergom	2	2	67	6	9	444
Nógrád				2	3	160
Pest	12	12	286	19	22	1369
Somogy	4	4	62	10	16	704
Szabolcs-Szatmár-Bereg	8	9	146	16	19	835
Tolna	3	3	77	6	6	322
Vas	7	7	162	5	6	368
Veszprém	4	4	101	8	10	558
Zala	5	5	121	6	8	312
Total:	122	125	2976	203	263	14196

“The entire facility must meet certain standards for us to consider it suitable for the program.”

#### Curriculum

The findings indicate that the program is strongly embedded in the Hungarian public education system and is closely aligned with national regulatory frameworks (29, 30). Swimming education is integrated into both preschool and primary school curricula, ensuring broad and equitable access at the institutional level. The structured 9-level system provides a standardized developmental pathway (Table 3), enabling continuous monitoring and comparability of children's progress across locations. This contributes to the system-level coherence of the program. At the same time, the lack of a fully unified national teaching methodology introduces variability in implementation, which may affect the consistency of learning outcomes. This suggests a tension between centralized program design and local-level pedagogical practices.

**Table 3:** 9 levels and requirements of Swimming National Programme. Source (33):.

Levels	Requirement
1. Tadpole	Practises for getting used to the water
2. Turtle	Tasks for acclimatization to deep water, basics of backstroke
3. Seahorse	Sole jumping, floating, swimming out. Backstroke up to 15 meters in deep water. Fast leg pace with a kickboard up to 15 meters.
4. Seal	Backstroke up to 25 meters and pedalling for 5 s.
5. Penguin	Basics of dive entry, standing jump, followed by pedalling for 15 s.
6. Ray fish	Breaststroke up to 25 meters.
7. Walrus	Freestyle at a basic level up to 10–15 meters. Water rescue.
8. Crocodile	Freestyle up to 25 meters.
9. Beaver	Knowledge and performance of three swimming styles up to 25–50 meters. Self-rescue from the water.

The curriculum’s 3 × 36 h are divided into the following 9 levels.

“Annually, children can realistically progress 2–3 levels, which justifies the structure of the 9-level system.”

#### Instructors

The availability and qualification of instructors emerged as one of the most critical factors influencing program effectiveness. While the program ensures that only licensed instructors can participate (31), the rapid expansion of the initiative has significantly increased demand for qualified professionals. The findings highlight regional disparities in instructor availability, as well as variability in training quality, which may impact the consistency of program delivery. At the same time, the existence of a multi-level supervision system contributes to maintaining professional standards and monitoring instructional quality. These results indicate that human resource capacity represents a key bottleneck in scaling the program sustainably at the national level.

“Finding enough well-prepared instructors is one of the biggest challenges of the program.”

#### Logistics

The logistics system represents one of the most distinctive and innovative components of the program. Centralized transportation ensures that children can access swimming facilities within a maximum travel time of 20 min, significantly enhancing accessibility and reducing territorial inequalities. This logistical framework plays a crucial role in enabling participation, particularly in areas where local infrastructure is insufficient. At the same time, it introduces a high level of organizational complexity and dependency on external operational conditions, such as transport capacity, scheduling, and institutional coordination. Thus, logistics can be interpreted as both an enabling mechanism and a potential vulnerability within the system.

“The key principle is that the swimming pool must be accessible within 20 min.”

#### Talent development

Although the primary objective of the program is swimming education and water safety, talent identification and development emerge as important secondary outcomes. The introduction of the Bridge Program creates a structured pathway between mass participation and competitive sport. The findings suggest that the program contributes to expanding the talent pool by exposing a large number of children to swimming at an early age, thereby increasing the likelihood of identifying potential elite athletes. This dual function—mass participation combined with talent identification—positions the program within a broader sport development framework.

“The goal is that talented children continue in competitive sport after the program.”

#### Evaluation and future directions

The analysis identified a complex system characterized by both significant strengths and structural challenges, which are summarized in a SWOT framework (Table 4). Among the strengths, the nationwide reach, free access, and the integrated nature of infrastructure, education, and logistics stand out. This level of systemic integration enables large-scale participation and contributes to the social and educational impact of the program. At the same time, several challenges limit its long-term sustainability. The shortage of qualified instructors, infrastructural constraints, and organizational issues—such as cancellations due to institutional factors—represent persistent barriers. These challenges are closely linked to the program's rapid expansion and its dependence on human and infrastructural resources. In addition, the need for further development of IT systems and data management processes highlights the increasing importance of digital infrastructure in supporting large-scale programs and ensuring efficient monitoring and evaluation. The SWOT analysis further indicates that the program holds significant opportunities in expanding participation and talent identification, while also facing external threats related to resource availability and system capacity (Table 4). Overall, the findings suggest that while the program is highly complex, this complexity is both its primary strength and a source of operational challenges.

**Table 4:** SWOT analysis of the Swimming Nation Program, source: own-edition.

Strengths	Weaknesses
• A nationwide increase in infrastructure investments and the construction of swimming pool facilities. • Positive effects of swimming education and health promotion in the participating age groups. • Free access for children enrolled in the program, including transportation, instruction, and equipment. • Contribution to job creation. • Improved utilization of swimming pool facilities. • Recognition and strengthening of the Hungarian swimming education system and its sports professionals.	• Methodological inconsistencies among existing instructors at the national level. • Regional disparities in the number of available instructors. • A shortage of instructors trained in swimming education for children with disabilities. • Inadequate conditions of certain existing swimming facilities that may hinder the program's effectiveness. • Capacity and equipment limitations in some existing swimming pools.
**Opportunities**	**Threats**
• Acquisition of water safety skills and swimming proficiency within the targeted age groups. • Identification and selection of talent for Hungarian competitive swimming from a broader youth base. • Talent identification for multiple aquatic sports from an expanded pool of children. • Integration of children with disabilities into groups through structured swimming education. • Introduction of a wider range of aquatic sports within swimming pool facilities. • Development and modernization of deficiencies in previously constructed swimming facilities.	• A potential decline in public swimming activities in swimming facilities participating in the program. • Difficulties in recruiting a sufficient number of qualified swimming instructors. • Limited availability of swimming instructors trained within a high-quality educational system. • Challenges in ensuring the availability of bus drivers with an appropriate pedagogical attitude. • Insufficient number of swimming facilities that meet the quality requirements of the program.

“The program is highly complex, but this complexity is also its greatest strength.”

## Discussion and conclusions

The present study set out to examine how the Swimming Nation Program operates in practice, with particular attention to its structural components and implementation mechanisms. Based on the combined analysis of documents and expert interviews, it appears that the program functions as a coordinated system in which infrastructure, educational content, human resources, and organizational processes are closely connected. One of the more important findings is that the program does not serve a single purpose. While its primary objective is clearly related to swimming education and water safety, it also creates conditions that may facilitate talent development. By reaching a large number of children at an early age, the program increases the likelihood that those with higher potential will enter competitive pathways later on. This observation is consistent with long-term development models, which emphasize that a broad participation base is often a prerequisite for elite performance (8, 9).

At the same time, the results draw attention to the fact that structural conditions continue to play a decisive role. Access to appropriate facilities is not evenly distributed, and this directly affects where and how the program can be implemented. Similar patterns have been reported in other youth sport systems, where infrastructural background strongly influences participation opportunities (24, 25). In this respect, the effectiveness of the program cannot be evaluated independently of the material environment in which it operates. The role of logistics deserves particular attention. The findings suggest that transportation is not simply an operational detail but a central element of the system. In many cases, participation would not be possible without organized transport, especially in areas lacking suitable facilities. This highlights that accessibility is shaped not only by the presence of infrastructure but also by the ability to connect participants to it in a reliable way.

Human resources represent another area where challenges become visible. Although the program requires qualified instructors, the expansion of the system has increased demand to a level that is not always easy to meet. Differences between regions in terms of instructor availability and preparedness may influence the quality and consistency of implementation. Previous studies have similarly emphasized the importance of well-trained professionals in maintaining the effectiveness of youth development systems (24). From this perspective, the issue is not only quantitative (number of instructors) but also qualitative (training, methodological consistency). The analysis also points to a more general tension between centralized planning and local realization. The program is built on a clearly defined national framework, yet its implementation inevitably depends on local conditions. Differences in infrastructure, institutional cooperation, and professional practices can lead to variation in outcomes. This is not necessarily a weakness, but it does indicate that a certain level of flexibility is unavoidable in large-scale systems.

Looking at the program from a broader perspective, it can be seen as part of a wider shift in sport policy, where educational, health-related, and performance-oriented goals are increasingly addressed within the same framework. The Swimming Nation Program reflects this approach by combining elements of mass participation with pathways toward competitive sport. At the same time, its operation shows that such integration requires continuous coordination between different subsystems. Despite its strengths, several factors may limit the long-term sustainability of the program. The uneven distribution of infrastructure, the shortage of qualified instructors, and the growing importance of digital and organizational support systems all represent areas where further development may be needed. These challenges are closely linked to the scale of the program and the resources required to maintain it.

Certain limitations of the present study should also be noted. The relatively small number of interview participants and the focus on expert perspectives mean that the findings primarily reflect the views of those involved in program management and implementation. In addition, access to some internal documents was restricted, which may have influenced the depth of the analysis. Future research could broaden the scope by including other stakeholder groups, such as teachers, parents, or participants, and by examining the program's outcomes in more detail.

In summary, the Swimming Nation Program appears to be a structured and ambitious initiative, the effectiveness of which depends largely on how well its key components are aligned in practice. The interaction between infrastructure, logistics, and human resources seems to be particularly important in this regard. The experiences associated with the program may offer useful insights for the design and implementation of similar large-scale sport development initiatives, while also highlighting the need for ongoing adaptation to structural and organizational constraints.

## Data Availability

The original contributions presented in the study are included in the article/Supplementary Material, further inquiries can be directed to the corresponding author.
